# Brain Drain: Post Graduation Migration Intentions and the influencing factors among Medical Graduates from Lahore, Pakistan

**DOI:** 10.1186/1756-0500-4-417

**Published:** 2011-10-17

**Authors:** Nazish Imran, Zahra Azeem, Imran I Haider, Naeem Amjad, Muhammad R Bhatti

**Affiliations:** 1 Assistant Professor Child & Family Psychiatry Department. King Edward Medical University/Mayo Hospital Lahore, Pakistan; 2 House Officer, Surgery King Edward Medical University/Mayo Hospital Lahore, Pakistan; 3 Associate Professor. Department of Psychiatry& Behavioral Sciences. Fatima Memorial College of Medicine and Dentistry, Lahore, Pakistan; 4 Postgraduate trainee, Department of Psychiatry& Behavioral Sciences King Edward Medical University/Mayo Hospital Lahore, Pakistan; 5 Professor & Chairman Department of Psychiatry& Behavioral Sciences King Edward Medical University/Mayo Hospital Lahore, Pakistan

## Abstract

**Background:**

The increasing migration of health professionals to affluent countries is not a recent phenomenon and has been addressed in literature. However the various facets of physician migration from Pakistan, the third leading source of International medical graduates has not been rigorously evaluated. The objective of the current study was to survey final year students and recent medical graduates in Lahore, Pakistan about their intentions to train abroad, their post training plans as well as to identify the factors responsible for their motivation for international migration.

**Method:**

A self administered structured questionnaire was developed to collect respondents' demographic and educational characteristics, intention to train abroad, their preferred destination & post training intentions of returning to Pakistan. Various influencing factors which impact on medical graduate's motivation to train abroad or stay in Pakistan were explored using a 10 point scale. SPSS software was used for data entry and analysis.

**Results:**

Of the 400 eligible respondents, 275 responded (response rate 68.7%). One hundred and sixty six respondents (60.4%) intended to train abroad either for a specialty (54.9%) or a subspecialty (5.5%) The United States and United Kingdom were the most preferred destination. While 14.2% intended to return to Pakistan immediately after training, a significant percentage (10%) never intended to return to Pakistan or wished to stay abroad temporarily (37%). Professional excellence and establishing quickly in the competitive market were the most important goal to be achieved by the respondents for intention for postgraduate training abroad. The most common reasons cited for training abroad were the impact of residency training on future career (mean score 8.20 ± 2.3), financial conditions of doctors (mean score 7.97 ± 2.37) and job opportunities (mean score7.90 ± 2.34).

**Conclusion:**

An alarming percentage of medical graduates from Lahore, Pakistan intend to migrate for post graduate training, mainly to United States. A significant proportion wished to stay in the host country either temporarily or indefinitely. Impact of residency abroad on future career, financial conditions of doctors, job opportunities and better working conditions were some of the motivating factors behind the migration.

## Background

The increasing migration of international medical graduates from developing countries to more developed countries, a phenomenon known as "brain drain" has continued to fuel the huge inequities in global health. Around 23-28% of doctors working in the four major recipient countries (i.e. USA, UK, Canada and Australia) are International Medical graduates (IMG) and lower income countries supply 40-75% 0f these IMGs [1]. Pakistan is the third leading source of IMGs in the affluent countries [2]. The emigration ratio in Pakistan (the number of physicians working abroad as a percent of total pool of physicians) is estimated to be as high as 13.5%-17.6% [3].

Migration although has significant impact on all source countries [2,4] but for Pakistan with an anticipated shortfall in the year 2020 of 58,000 to 451,000 physicians [3] it has serious consequences. Pakistan ranks 129^th ^of the 174 nations on the Human Development Index, a measure of the achievement of a country in terms of health, longevity, education and standards of life"[5]. It is struggling with double burden of disease; an ongoing burden of infectious diseases and malnourishment while the cardiovascular disease, cancer etc are also on the rise. Because of migration of health professionals, Pakistan loses intellectual capital and educational investment. When émigrés leave, people who can resume role of leaders to reform & stabilize the health system and drive changes in academics and research are lost thus putting enormous pressure on an already vulnerable healthcare system.

Migration of physicians from many low [1,2,6] and middle income countries[7] has been addressed in literature but little has been published about such migration from Pakistan. We were interested in exploring the dynamics of physician migration from Lahore, Pakistan. As residency training abroad is a critical and initial step in Physician migration abroad, the objective of our study was to survey group of final year medical students and house officers about their intentions to train abroad and their post training plans. We also wish to highlight the major push and pull factors behind this brain drain.

## Methods

### Setting

The study was conducted in King Edward Medical University (KEMU) in Lahore, the second largest city of Pakistan. King Edward Medical University is the oldest medical Institution in Pakistan. Students achieving highest merit in the Province of Punjab (on the basis of academic record and on entrance test) get admission to this prestigious institution and as such represents the brightest among the medical students. Institutional review board of KEMU approved the study and informed consent was obtained from all participants. Our target population consisted of final year students and house officers as this is the typical period during which they decide regarding their post graduation plans.

### Questionnaire

A structured self administered questionnaire was developed based on previous studies done on this topic as well as discussion and input from the medical students. We pilot tested the questionnaire with 10 post graduate trainees who recently started their training. The questionnaire was in English language as it is the main language in medical education in Pakistan.

The questionnaire had various sections. First section collected demographic information (age, gender, marital status, socio economic conditions) and educational characteristics (designation, planned residency type). Second part asked about respondents' intentions for post graduate training abroad, the destination country and post training intent in terms of returning or not to Pakistan.

Various influencing factors which impact on medical graduate's motivation to train abroad or stay in Pakistan were explored next. The questionnaire used a 10 point scale.

Additional questions about attitudes towards conditions in Pakistan and prospects abroad were also asked (results not reported here).

The survey questionnaire was anonymous and confidential. It was distributed at the end of final year class as well as during visits to all the units in Mayo hospital and collected later by the investigators.

### Analysis

Data was entered and analyzed by SPSS 17. 0. Descriptive statistics were presented of demographic and educational characteristics and the outcome variable of interest (i.e. intention to migrate abroad) using frequency and percentage for categorical variables and mean and standard deviation for continuous variables. Chi square test was used to determine association between intention to migrate abroad for post graduation and various factors. For all purposes, P value <.05 was considered as significant.

## Results

Of 400 eligible participants, 275 responded to the survey (response rate 68.7%). The mean age of the sample was 23.67(S.D 3.45) with almost half being male (51.3%) and majority were single (72%). Most of the respondents (95.3%) were Muslim. 153(55.6%) were final year students and almost 60% belonged to urban area. Majority of students belonged to upper middle class (64.7%).

Medicine was the planned residency by 54.5% of respondents followed by surgery (21.8%) while 8% intended to go directly to practice without completing residency training.

One hundred and sixty six respondents (60.4%) intended to train abroad either for a specialty (54.9%) or a subspecialty (5.5%) while 10% were not clear about their future intentions. (Table 1) The United States and United Kingdom were the most preferred destination. While 14.2% intended to return to Pakistan immediately after training, a significant percentage (10%) never intended to return to Pakistan or wished to stay abroad temporarily (37%).

**Table 1:** Respondents post-graduation migration intentions & post training plans.

	N (%)
**Plan to train abroad (N = 270)**	
*No*	77(28.0)
*Yes, for specialty training*	151(54.9)
*Yes. For subspecialty training*	15(5.5%)
*Don't know*	27(9.8)

**Preferred training country (N = 184)**	
*USA*	96(34.9)
*United Kingdom*	44(16.0)
*Australia*	19(6.9)
*Gulf countries*	10(3.6)
*Canada*	9(3.3)
*Others*	6(2.2)

**Post abroad training plan (N = 166)**	
*Return directly to Pakistan*	39(14.2)
*Work abroad <5 years then return*	27(9.8)
*Work abroad 5-10 years then return*	48(17.5)
*Work abroad > 10 years then return*	26(9.5)
*Never return to Pakistan*	26(9.5)

**Figure 1:**
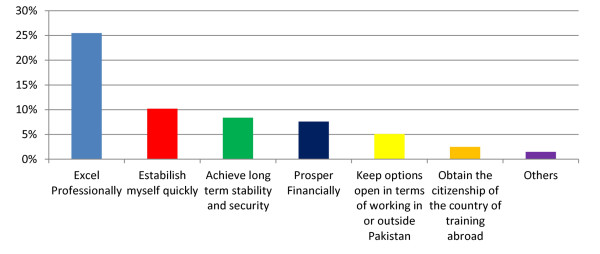
**Respondents most important goal to be acheived through training abroad**.

Professional excellence and establishing quickly in the competitive market were the most important goal cited by the respondents to be achieved through postgraduate training abroad (Figure 1).

The most common reason cited for training abroad was the impact of residency training on future market(mean score 8.20 ± 2.3). Another important issue concerned the poor salary of the postgraduate trainees in PGME (post graduate medical education) in Pakistan and working conditions for residents and doctors alike. (Table 2) Job opportunities, Quality of clinical & research training was also cited as an extremely important reason behind respondents decision to migrate. All other factors and mean scores for various factors are given in Table 2. Political conditions in the country was considered as having least contribution to the decision of respondents to migrate (mean 5.72 ± 2.89).

**Table 2:** Respondents Reasons for Migration for Post-Graduation or Staying In Pakistan.

	**Mean (standard deviation**)
**Reasons for migration to train abroad**	
*1. Impact of residency training on future career*	8.20(2.35)
*2. Financial conditions of doctors*	7.97(2.37)
*3. Job opportunities*	7.90(2.34)
*4. Financial situation of residents*	7.9(2.41)
*5. Working conditions of residents*	7.89(2.34)
*6. Working condition of doctors*	7.87(2.29)
*7. Clinical training*	6.98(2.51)
*8. Research Training*	6.93(2.74)
*9. Teaching in Residency Programs*	6.63(2.74)
*10. Social conditions*	6.39(2.86)
*11. Residency training opportunities*	6.35(2.7)
*12. Personal conditions*	5.94(2.84)
*13. Political conditions*	5.72(2.89)

**Reasons for staying in Pakistan for Post graduation**	
*1. Family ties in Pakistan*	8.17(2.60)
*2. Desire to settle in Pakistan*	7.64(2.67)
*3. Desire to serve our people/nation*	7.57(2.71)
*4. Religious factors*	6.38(2.85)
*5. Professional satisfaction*	6.08(2.61)
*6. Adequate/alternative financial support*	5.68(2.78)
*7. Lack of necessary resources.*	5.26(3.08)
*8. Quality of training in Pakistan*	5.18(2.20)
*9. Political factors.*	4.75(3.26)
*10. Visa Problems*.	4.37(2.81)

The major factors for students who wished to stay in Pakistan were family ties, desire to settle in Pakistan and serve our population. Professional satisfaction due to large number of patients encountered with vast variety of presentations and hands on clinical experience also contributed significantly among reasons to stay in Pakistan.

Chi square analysis between various factors (age, marital status, gender, address & socioeconomic conditions) and plan to train abroad revealed only marital status having significant association (P value < .05).

Responses to open ended question revealed that increase bulk of patients and exposure to a wide variety of cases & being close to family were the main strengths of training in Pakistan identified by respondents. Poor working environment (bullying attitudes of seniors, less positive feedback for good work), poor salary structure and long working hours were considered as some of the weaknesses in training in Pakistan. Better pay and working conditions and improvement in quality of training and research opportunities were the suggested changes by the respondents which would influence them to consider further training in Pakistan.

## Discussion

We found that 166(60.4%) of the medical students and house officers responding to our survey intended to travel abroad for specialty training (54.9%) or subspecialty training(5.5%). These results are in line with survey of 166 final year students of Indian medical schools in 2004 where 59% thought of training abroad [8]. Another study concluded that about one half of South African medical graduates migrate [9]. On the other hand this percentage is lower than that reported in studies from Lebanon (96%) [7]as well as in the only published study from Pakistan in two private medical Institutions of Karachi where 95% and 65% intended to train abroad [10]. We feel the difference may be because of difference in socio economic background of the two groups Medical graduates belonging to private medical colleges (which have a higher tuition fee in Pakistan) who are likely to belong to more affluent and financially sound families have the resources and the contacts to train abroad.

The intention to migrate abroad of significant proportion of health professionals is of concern and threatens the ability of Pakistan to meet the health care needs of their own population. As migratory flows may increase in future, [11] policy initiatives are needed to counter the effects on local healthcare sector.

Top destination for our respondents are the same as reported previously in literature on this topic, i.e. United States, United kingdom, Australia, Gulf countries and Canada. Recently trend of migration towards Gulf countries appears to have increased perhaps due to Memorandum of understanding signed between the health ministry of Saudi Arabia and College of Physicians & Surgeons (The post graduate training authority) in Pakistan and facilitation of the process by the Pakistani Government.

Desire to settle abroad to more affluent nations is generally considered and assumed to be the main motivation of people who migrate abroad. However we found that only around 10% of our respondents wanted to settle abroad but would rather prefer to either return directly to Pakistan or stay temporarily abroad before returning to Pakistan. This implies that we need to address the factors that compel the medical health professionals to migrate abroad in the first instance and also those which may lead to change in their intentions to return or settle in Pakistan. A small study from Pakistan showed people funded for a doctorate returning and on return facing major non financial disincentives for good performance [12]. Also many doctors who do return with high technical skills find very few satisfying jobs available to them which further acts as a barrier in their return to homeland. Thus brain drain is compounded by the fact that emigrating skilled workers are more likely to stay in their host country.

Results of the study showed that respondents wish to migrate due to impact of training abroad on future career, to gain competitive advantage in the saturated job market, as well as to have financial security, better working conditions and a better training experience. This study results fit well with previous studies which have cited financial factors, poor working conditions, heavy workload, lack of training opportunities, a lack of sufficient opportunities for promotion etc [10,13,14] as motivating factors for migration.

Salary of Postgraduate medical students in Pakistan is very poor. Availability of few training positions for number of medical graduates means many people work without pay to gain required experience for examinations. Similarly working conditions in post graduate medical education (PGME) In Pakistan leaves much to be desired. Students intend to migrate not only for monetary gains but also because of high prevalence of bullying [15] and to escape the hierarchical system which is in place in majority of medical Institutions in Pakistan.

The complex issue of migration of medical graduates does not have a readymade solution. All the stake holders i.e the Governments of the source country and the host country, professional unions, International bodies, and medical workforce in the country need to focus and take some bold steps to prevent or decrease unwanted migration of bright students from the country. Partnerships between Institutions in developed and developing countries are needed to encourage doctors to return [16]. Review of pay structure, improvement in quality of training in Pakistan and making work environment more conducive to post graduate trainees are some of the steps which may help in dealing with physicians' migration.

In terms of limitations our study had a small sample size and involved only one site thus results may not be generalizable however we hope that our study has yielded insights into factors responsible for doctors migration from Pakistan. Strengths of the study included good response rate as well as being the only study from Pakistan which along with respondents' plans to train abroad also assessed their post-training migration intentions.

## Conclusion

The findings of our study are of concern in terms of future migration of our medical graduates and have implications for healthcare and academic policies. It has also highlighted various facets of migration of doctors from Pakistan. Unless steps are taken urgently to address the factors contributing to the migration of doctors, the brain drain will continue to fuel huge inequalities in global health.

## Abbreviations

(IMG): International Medical graduates; (PGME): Post graduate medical education

## Competing interests

The authors declare that they have no competing interests.

## Authors' contributions

**NI**: Conception and design, data analysis & interpretation, article drafting. **ZA**: conception and design, data collection, analysis. **IIH**: design, article drafting, critical revision. **NA**: Data collection, critical revision. **MRB**: design, critical revision. All the authors read & approved the final draft of the article.

## References

[B1] Chen LC Bouffard JI Fatal flows-doctors on the move N Engl J Med 2005 353 17 1850 2 10.1056/NEJMe058188 16251543

[B2] Mullan F The metrics of the physician brain drain N Engl J Med 2005 353 1810 1818 10.1056/NEJMsa050004 16251537

[B3] Talati JJ Pappas G Migration, Medical Education, and Health Care: A View from Pakistan J Acad Med 2006 81 12 Suppl S55 S62 10.1097/01.ACM.0000243543.99794.07 17086048

[B4] Hagopian A Ofusus A Fatusi A The flight of physicians from West Africa: views of African physicians and implications for policy Soc Sci Med 2005 61 1750 1760 10.1016/j.socscimed.2005.03.027 15927335

[B5] Haq M Human Development in South Asia 2000 Karachi: Oxford University Press

[B6] Adams O Kinnon C A public health perspective. International trade in health services: a developmental perspective 1998 Geneva, World Health Organisation

[B7] Akl EA Maroun N Major S Afif C Abdo A Choucair J Post graduation migration intentions of students of Lebanese medical schools: a survey study BMC Public Health 2008 8 191 10.1186/1471-2458-8-191 18518954 PMC2424042

[B8] Rao NR Rao UK Cooper RA Indian medical student' views on immigration for training and practice Academic Medicine 2006 81 2 185 88 10.1097/00001888-200602000-00020 16436584

[B9] Dambisya Y Career intentions of UNITRA medical students and their perceptions about the future Education for health 2003 16 3 286 297 10.1080/13576280310001607442 14741877

[B10] Syed NA Khimani F Andrades M Ali SK Paul R Reasons for migration among medical students from Karachi Medical Education 2008 42 61 68 18042189 10.1111/j.1365-2923.2007.02904.x

[B11] Brown RP Connell J The migration of doctors and nurses from South Pacific Island Nations Social Sciences & Medicine 2004 58 2193 2210 10.1016/j.socscimed.2003.08.020 15047077

[B12] Oman KM Moulds R Usher K Specialist training in Fiji: Why do graduates migrate, and why do they remain? A qualitative study Human Resources for health 2009 7 9 10.1186/1478-4491-7-9 19216766 PMC2652983

[B13] Cooper RA Physician migration: a challenge for America, a challenge for the world J Contin Educ Health Prof 2005 25 8 14 10.1002/chp.3 16078797

[B14] Hyder AA Akhtar A Qayyum A Evaluating capacity development for health research in Pakistan: case study on doctoral training Health Pol Plan 2003 18 338 43 10.1093/heapol/czg040 12917275

[B15] Imran N Jawaid M Haider II Masood Z Bullying of junior doctors in Pakistan: a cross-sectional survey Singapore Med J 2010 51 7 592 5 20730401

[B16] Patel V Recruiting doctors from Poor countries: the great brain robbery? BMJ 2003 327 926 8 10.1136/bmj.327.7420.926 14563760 PMC218826

